# Emerging Therapeutic Targets in Oxaliplatin-Induced Acute Peripheral Neuropathy: Biochemical Protection by Organoselenium via Redox and Ionic Regulation

**DOI:** 10.1021/acs.chemrestox.6c00302

**Published:** 2026-08-18

**Authors:** Vanessa M. E. da Rocha, Ketlyn P. da Motta, Ana Paula B. Wille, Ariana S. Lima, Diéssica Padilha Dalenogare, Vinicius Costa Prado, Diego Alves, Angélica S. Reis, Ethel A. Wilhelm

**Affiliations:** † Preclinical and Translational Research Group on Pain and Chronic Diseases − GPCCD, 37902 CCQFA − Federal University of Pelotas, UFPel, P.O. Box 354, Pelotas, Rio Grande do Sul 96010-900, Brazil; ‡ Laboratory of Clean Organic Synthesis − LASOL, CCQFA - Federal University of Pelotas (UFPel), P.O. Box 354, Pelotas, Rio Grande do Sul 96010-900, Brazil

## Abstract

Oxaliplatin (OXA), a platinum-based chemotherapeutic agent, frequently induces acute peripheral neuropathy characterized by mechanical and cold allodynia, for which effective therapeutic options remain limited. Here, we investigated the acute antinociceptive and biochemical effects of 4-amino-3-(phenylselanyl)­benzenesulfonamide (4-APSB), a compound with antioxidant and antinociceptive properties, in female Wistar rats with OXA-induced acute neuropathy. Animals received OXA (4 mg/kg, i.p.) on two consecutive days and were subsequently treated with 4-APSB (1 mg/kg, i.g.) or duloxetine (30 mg/kg, i.g.) as a reference drug. Nociceptive behaviors were assessed using mechanical and cold sensitivity assays, followed by biochemical analyses in nervous tissues. OXA administration induced mechanical and cold hypersensitivity, elevated oxidative stress markers, and disrupted membrane enzyme function, reducing Na^+^/K^+^-ATPase activity and increasing acetylcholinesterase (AChE) activity. Treatment with 4-APSB significantly attenuated hypersensitivity, increased Na^+^/K^+^-ATPase and catalase activities, reduced reactive species generation, and attenuated the OXA-induced increase in AChE activity in the cerebral cortex, without affecting locomotor behavior. Collectively, these findings show that 4-APSB produces acute antinociceptive effects accompanied by the modulation of redox homeostasis, Na^+^/K^+^-ATPase activity, and AChE activity following OXA exposure. The coordinated improvement in behavioral and biochemical parameters supports the potential relevance of redox balance and membrane-associated enzyme function to the pharmacological effects of 4-APSB. These results identify 4-APSB as a promising compound for further investigation as a therapeutic strategy for OXA-induced peripheral neuropathy.

## Graphical abstract

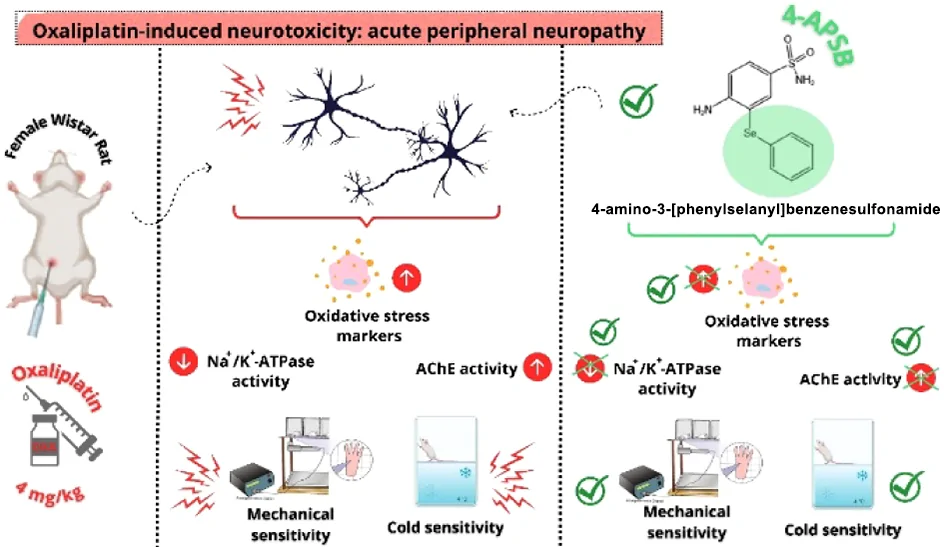

## 1 Introduction

Platinum-based antineoplastic agents are widely used to treat solid tumors such as colorectal, gastric, breast, and head and neck cancers.^1^ This class of chemotherapeutic drugs, including oxaliplatin (OXA), is well known for its neurotoxicity, which is often associated with neuropathic pain.^1,2^

Chemotherapy-induced peripheral neuropathy (CIPN) is a frequent and heterogeneous complication of chemotherapy, typically presenting with bilateral sensory symptoms, such as pain, numbness, and weakness. Its prevalence can reach 68% in the first month and about 30% chronically.^3^ Moreover, CIPN is a dose-limiting adverse effect of antineoplastic therapy, impairing quality of life and often requiring treatment modification or discontinuation.^2^

The primary mechanism underlying OXA-induced acute peripheral neuropathy involves the dysfunction of voltage-gated sodium channels caused by oxalate metabolites, which disrupt channel activity in sensory neurons of the dorsal root ganglion, leading to neuronal hyperexcitability.^4^ In addition, impairments in mitochondrial function, including bioenergetic failure and increased production of reactive oxygen species, have also been implicated.^1^

Using preclinical rodent models, studies from our research group have demonstrated that OXA inhibits activity and reduces the expression of Na^+^/K^+^-ATPase, suggesting that its modulation may be a promising approach against OXA-induced neuropathy.^5,6^ We also observed that OXA increases acetylcholinesterase (AChE) activity in the cerebral cortex, hippocampus, and spinal cord, indicating a possible link between this enzyme and neuropathic pain.^7^ Despite these advances, the mechanisms of OXA-induced neurotoxicity are still not fully understood, and there are no specific medications approved for its treatment.^8^

Current therapeutic options for neuropathic pain include tricyclic antidepressants (TCAs), serotonin and norepinephrine reuptake inhibitors (SNRIs), anticonvulsants, and opioid agonists.^3^ Among available neuropathic pain treatments, only the SNRI duloxetine, FDA-approved for depression and diabetic neuropathy, has shown moderate efficacy in CIPN, with no comparable alternatives offering a favorable risk–benefit balance.^3^

However, although several drugs show promising results in preclinical models, few have demonstrated efficacy in clinical studies, reinforcing the need for new therapies.^8^ Within this framework, 4-amino-3-(phenylselanyl)­benzenesulfonamide (4-APSB) stands out. This molecule has been investigated by our research group for its antioxidant action and antinociceptive effects, both central and peripheral, at low doses.^9^ It also attenuates fibromyalgia induced by intermittent cold stress in mice.^10^

Taken together, these findings highlight 4-APSB as a promising candidate for managing chemotherapy-induced neuropathy. Building on previous evidence of its antioxidant and antinociceptive properties, the present study aimed to evaluate its protective effects against acute OXA-induced neurotoxicity in female rats.

## 2 Materials and Methods

### 2.1 Animals

Female Wistar rats (200–300 g) bred in-house and maintained at a controlled temperature (22 ± 2 °C) under a light/dark cycle of 12:12 h (lights on from 7:00 am to 7:00 pm), were used. The animals were accommodated in ventilated cages (10 per cage) with wood shaving bedding and nesting material, with free access to water and food. All experiments were conducted in accordance with the guidelines of the Ethics Committee on the Use of Animals of the Federal University of Pelotas (UFPel) (CEEA4506-2017). Behavioral studies followed the Animal Research: Reporting of In Vivo Experiments (ARRIVE) guidelines.^11^ All experiments were performed by an operator blinded to drug administration. Also, experimenters were blinded to the experimental group during the analysis.

### 2.2 Drugs

Compound 4-APSB (Figure 1) was synthesized and characterized at the Clean Organic Synthesis Laboratory (LASOL) at UFPel, according to Sacramento et al..^9^ The GC/MS analysis confirmed its chemical purity (99.9%). 4-APSB was dissolved in canola oil and administered intragastrically (i.g.) at a constant volume of 10 mL/kg body weight. All other chemicals used in this study were of analytical grade and obtained from standard commercial suppliers.

**Figure 1:**
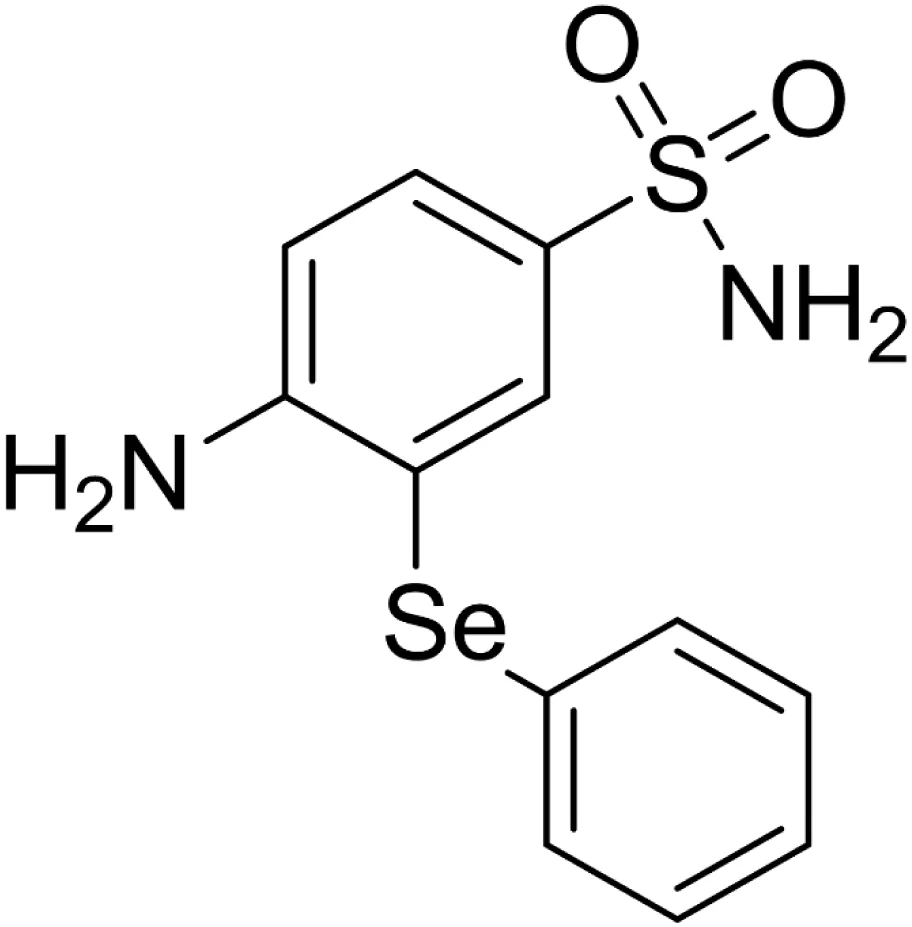
Chemical structure of 4-amino-3-(phenylselanyl)­benzenesulfonamide (4-APSB).

OXA was dissolved in a 5% glucose solution, administered intraperitoneally (i.p.), and was purchased from the company Eurofarma (São Paulo, Brazil). All other chemicals used in this study were of analytical grade and obtained from Sigma-Aldrich (St. Louis, MO, USA).

### 2.3 Experimental Design

Peripheral neuropathy was induced by OXA following a previous experimental protocol.^12^ Animals were randomly assigned to five experimental groups (8–9 animals/group): (i) control + vehicle, (ii) control + 4-APSB, (iii) OXA-induced + vehicle, (iv) OXA-induced + 4-APSB, and (v) OXA-induced + duloxetine. On days 1 and 2, animals in the OXA, OXA + 4-APSB, and OXA + duloxetine groups received OXA intraperitoneally (4 mg/kg, i.p.), while the animals in the control and 4-APSB groups received vehicle (5% glucose solution, 10 mL/kg, i.p.).

On day 2, 0.5 h after OXA administration, animals in the OXA + 4-APSB and 4-APSB groups received a single dose of 4-APSB (1 mg/kg, i.g.), and the animals in the OXA + Duloxetine group received Duloxetine (30 mg/kg, i.g.), while the animals in the Control and OXA groups received Vehicle (canola oil, 10 mL/kg, i.g.).

Mechanical and thermal sensitivities were assessed before treatment (baseline values) and 0.5 h after the administration of 4-APSB, duloxetine, or vehicle to determine the sensitivity threshold. At the end of the experimental protocol, the animals were euthanized by isoflurane inhalation. Sciatic nerve, spinal cord, cerebral cortex, and cerebellum samples were rapidly dissected, weighed, and placed on ice, and subsequently used for ex vivo assays (Figure 2).

**Figure 2:**
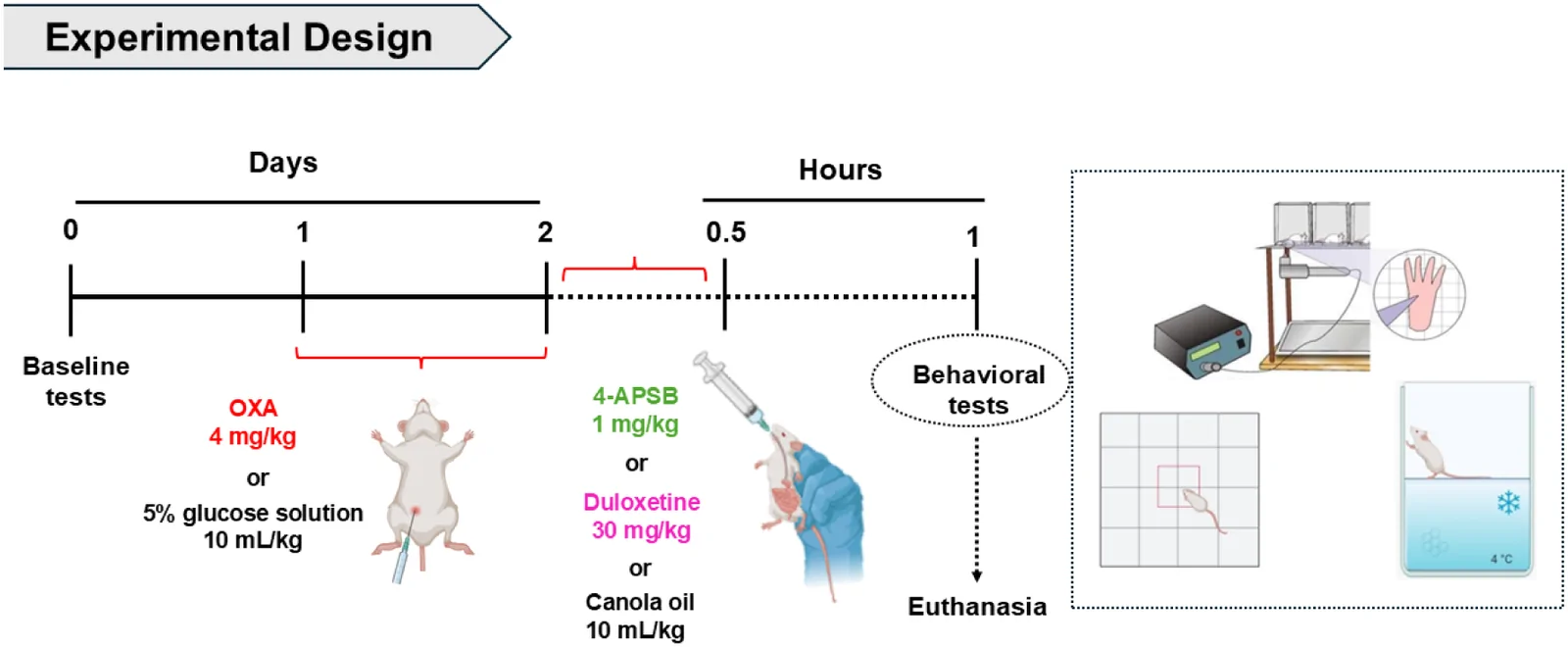
Summary of the experimental protocol for the induction of acute peripheral neuropathy in rats.

#### 2.3.1 Behavioral Tests

##### 2.3.1.1 Measurement of Mechanical Sensitivity

Mechanical sensitivity was evaluated as previously described.^13^ The test consisted of evoking hind paw flexion reflex using a portable force transducer (digital esthesiometer, Insight, São Paulo, Brazil) fitted with a polypropylene tip. The paw withdrawal threshold was determined by applying the tip perpendicularly to the mid-plantar surface of the hind paw with progressively increasing pressure until withdrawal occurred. The applied force was automatically recorded.

##### 2.3.1.2 Measurement of Thermal Sensitivity

Thermal sensitivity was assessed using the cold plate test, adapted from a previous protocol.^14^ Animals were individually placed on an acrylic plate maintained at 4 °C with ice for 5 min. The first response to the cold stimulus, defined as hind paw lifting, was recorded, along with the total number of responses during the test period.

##### 2.3.1.3 Assessment of Locomotor and Exploratory Domains

The open field test was conducted to evaluate general locomotor and exploratory behaviors.^15^ The apparatus consisted of plywood boxes with 30-cm-high walls and a 45 cm^2^ floor divided into nine squares (3 × 3) with masking tape. Each animal was placed in the center of the arena and observed for 4 min. Locomotor activity was quantified as the number of squares crossed with all four paws, while exploratory behavior was measured as the number of rearing events on the hind limbs.

#### 2.3.2 Ex Vivo Assays

##### 2.3.2.1 Na^+^/K^+^-ATPase and Mg^2+^-ATPase Activities

For the Na^+^/K^+^-ATPase activity assay, a reaction mixture was prepared containing S_1_, 3 mM MgCl_2_, 125 mM NaCl, 20 mM KCl, and 50 mM Tris-HCl, pH 7.4. The reaction was initiated by the addition of ATP to a final concentration of 3.0 mM. Control assays were performed under the same conditions with the addition of 0.1 mM ouabain. Samples were incubated at 37 °C for 30 min, and the incubation was stopped by adding a 10% trichloroacetic acid (TCA) solution containing 10 mM HgCl_2_. Na^+^/K^+^-ATPase activity was calculated as the difference between the two assays. Released inorganic phosphate (Pi) was measured according to a previous protocol.^16^ Enzyme activity was expressed as nmol of Pi/mg of protein/min.

For the Mg^2+^-ATPase activity assay, a reaction mixture was prepared containing S_1_, 3 mM MgCl_2_, 125 mM NaCl, 20 mM KCl, and 50 mM Tris-HCl, pH 7.4. Controls to correct for nonenzymatic substrate hydrolysis were prepared by adding sample preparations after the reactions were stopped with TCA. To determine the Mg^2+^-ATPase activity, we added ouabain (1 mM) to the reaction medium. The reaction was initiated by the addition of ATP and stopped after 30 min of incubation with 10% TCA. Enzyme activity was expressed as nmol of Pi/mg of protein/min.^16^

##### 2.3.2.2 Acetylcholinesterase (AChE) Activity

AChE activity was assayed based on the formation of the yellow 5,5′-dithio-bis-nitrobenzoic anion, measured spectrophotometrically at 412 nm for 2 min. The method was based on a previous protocol, with modifications, using acetylthiocholine as the substrate.^17^ The reaction mixture (final volume: 2 mL) contained S_1_ (100 μL), 100 mM K^+^-phosphate buffer (pH 7.5), and 1 mM 5,5′-dithio-bis-nitrobenzoic acid (DTNB). The enzyme was preincubated for 2 min at 25 °C. The reaction was initiated by the addition of 0.8 mmol/L acetylthiocholine iodide. The enzymatic activity was expressed as μmol/h/mg protein.

##### 2.3.2.3 Oxidative Stress Parameters

**Thiobarbituric acid reactive species (TBARS) assay.** The TBARS assay was performed to indirectly determine the malondialdehyde (MDA) levels, an important marker of lipid peroxidation. MDA reacts with 2-thiobarbituric acid (TBA) under acidic conditions and high temperatures to yield a chromogen, following a previously described protocol.^18^ S_1_ aliquots were incubated with 0.8% TBA, acetic acid buffer (pH 3.4), and 8.1% sodium dodecyl sulfate (SDS) for 2 h at 95 °C. The colorimetric reaction was measured at 532 nm, and results were expressed as nmol of MDA/mg of tissue.

**Reactive species (RS) levels.** RS levels were quantified using the 2’,7’-dichlorofluorescein diacetate (DCHF-DA) spectrofluorimetric assay.^19^ S_1_ (50 μL) was incubated with 20 μL of DCHF-DA (1 mmol/L) and 2430 μL of Tris-HCl (10 mmol/L, pH 7.4). The oxidation of DCHF-DA to fluorescent dichlorofluorescein (DCF) was measured to detect intracellular RS. DCF fluorescence intensity was recorded at 525 nm (excitation at 488 nm) 60 min after the addition of DCHF-DA. RS levels were expressed as arbitrary fluorescence units.

**Nitrates and nitrites (NOx) levels.** Nitrite and nitrate contents were measured to assess reactive nitrogen species levels using a previous method.^20^ The assay medium contained 2% VCl_3_ (in 5% HCl), 0.1% N-(1-naphthyl)­ethylenediamine dihydrochloride, and 2% sulfanilamide (in 5% HCl). After incubation at 37 °C for 60 min with an aliquot of S_2_, nitrite levels were determined spectrophotometrically at 540 nm, based on the VCl_3_-mediated reduction of nitrate to nitrite. The data were expressed as μmol of NOx/g tissue.

**Catalase (CAT) activity.** CAT activity was measured spectrophotometrically by monitoring H_2_O_2_ consumption at 240 nm.^21^ The enzymatic reaction was initiated by adding an aliquot of S_1_ (100 μL) to the substrate solution (H_2_O_2_, 105 μL, 0.3 mmol/L) in 50 mmol/L potassium phosphate buffer (pH 7.0). Activity was expressed in U/mg protein (1 U decomposes 1 mmol/L of H_2_O_2_ per minute at pH 7, 25 °C).

**Nonprotein thiols (NPSH) assay.** NPSH levels were determined as previously described.^17^ An aliquot of S_1_ was mixed (1:1) with 10% trichloroacetic acid (TCA) and centrifuged at 900*g* for 10 min. After centrifugation, the protein pellet was discarded, and free −SH groups were determined in the supernatant. The supernatant was added to 1 M potassium phosphate buffer (pH 7.4) containing 10 mM 5,5-dithiobis­(2-nitrobenzoic acid) (DTNB). The colorimetric reaction was measured at 412 nm. NPSH levels were expressed as μmol NPSH/g tissue.

##### 2.3.2.4 Protein Determination

Protein concentration was measured spectrophotometrically at 595 nm by the method of Bradford, using bovine serum albumin as the standard. This rapid and sensitive method quantifies microgram amounts of protein based on protein–dye binding. The reaction mixture contained S_1_ (50 μL) and Coomassie Brilliant Blue (2.5 mL) and was incubated for 10 min. Protein levels were expressed as mg protein/mL.^22^

### 2.4 Statistical Analysis

Statistical analysis was performed using GraphPad Prism 8.0 software (San Diego, CA, USA). Results are expressed as mean ± standard error of the mean (SEM). Data were analyzed by one-way analysis of variance (ANOVA), followed by Tukey’s post hoc test. Differences were considered statistically significant at *p* < 0.05.

## 3 Results

### 3.1 4-APSB Reduces OXA-Induced Mechanical and Thermal Hypersensitivities in Rats

The results presented in Figure 3(A-C) show that OXA treatment induced mechanical and thermal hypersensitivity in the animals. Specifically, there was a 37% decrease in the paw withdrawal threshold in the mechanical sensitivity test (Figure 3A) (F_(4, 42)_ = 192.4, *p* < 0.0001). In addition, OXA treatment caused a 52% reduction in the latency to the first nociceptive response to cold (Figure 3B) (ANOVA: F_(4, 40)_ = 16.36, *p* < 0.0001) and a 182% increase in cold sensitivity, as indicated by the total number of reactions (Figure 3C) (ANOVA: F_(4, 40)_ = 63.46, *p* < 0.0001), compared with the control group.

**Figure 3:**
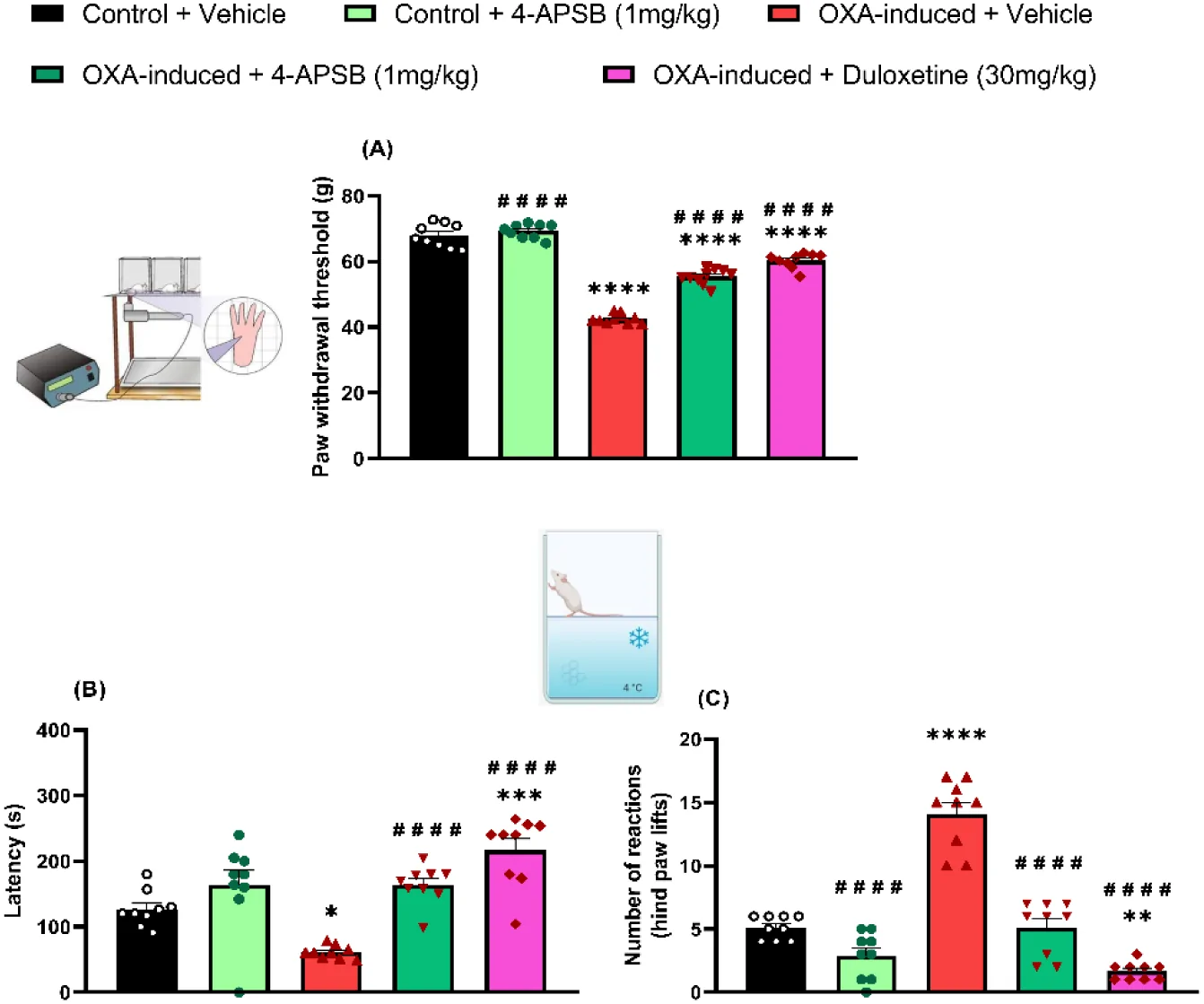
Effect of 4-amino-3-(phenylselanyl)­benzenesulfonamide (4-APSB) (1 mg/kg, i.g.) treatment on mechanical sensitivity (A) and thermal sensitivity: first reaction/nociceptive response to thermal stimulus (B) and total number of reactions to thermal stimulus (C) in a model of oxaliplatin (OXA)-induced acute peripheral neuropathy in female Wistar rats. The asterisks indicate significance levels when compared with the control group: (*) *p* < 0.05, (**) *p* < 0.01, (***) *p* < 0.001, and (****) *p* < 0.0001. (^####^) *p* < 0.0001 indicate significance levels when compared with OXA. One-way ANOVA followed by Tukey’s test.

Conversely, animals treated with 4-APSB (1 mg/kg) or duloxetine (30 mg/kg), used as the reference drug, showed a significant attenuation of OXA-induced hypersensitivity. Specifically, treatment with 4-APSB and duloxetine resulted in a 31% and 42% increase in the paw withdrawal threshold (Figure 3A), a 170% and 256% increase in the latency to the first nociceptive response to cold (Figure 3B), and a 64% and 88% decrease in cold sensitivity (Figure 3C), respectively, compared with the OXA-induced group. Moreover, OXA-induced animals receiving duloxetine exhibited a marked reduction in baseline cold sensitivity, as evidenced by a lower frequency of hind paw lifts (Figure 3B) and a prolonged withdrawal latency (Figure 3C) compared to the control group.

### 3.2 4-APSB Treatment Does Not Alter the Locomotor or Exploratory Activities of Rats

The results presented in Figure 4A,B show that 4-APSB treatment did not cause any significant changes in the number of crossings (ANOVA: F_(4, 30)_ = 1.644, *p* = 0.1890) or rearings (ANOVA: F_(4, 30)_ = 1.768, *p* = 0.1614) in the open field test.

**Figure 4:**
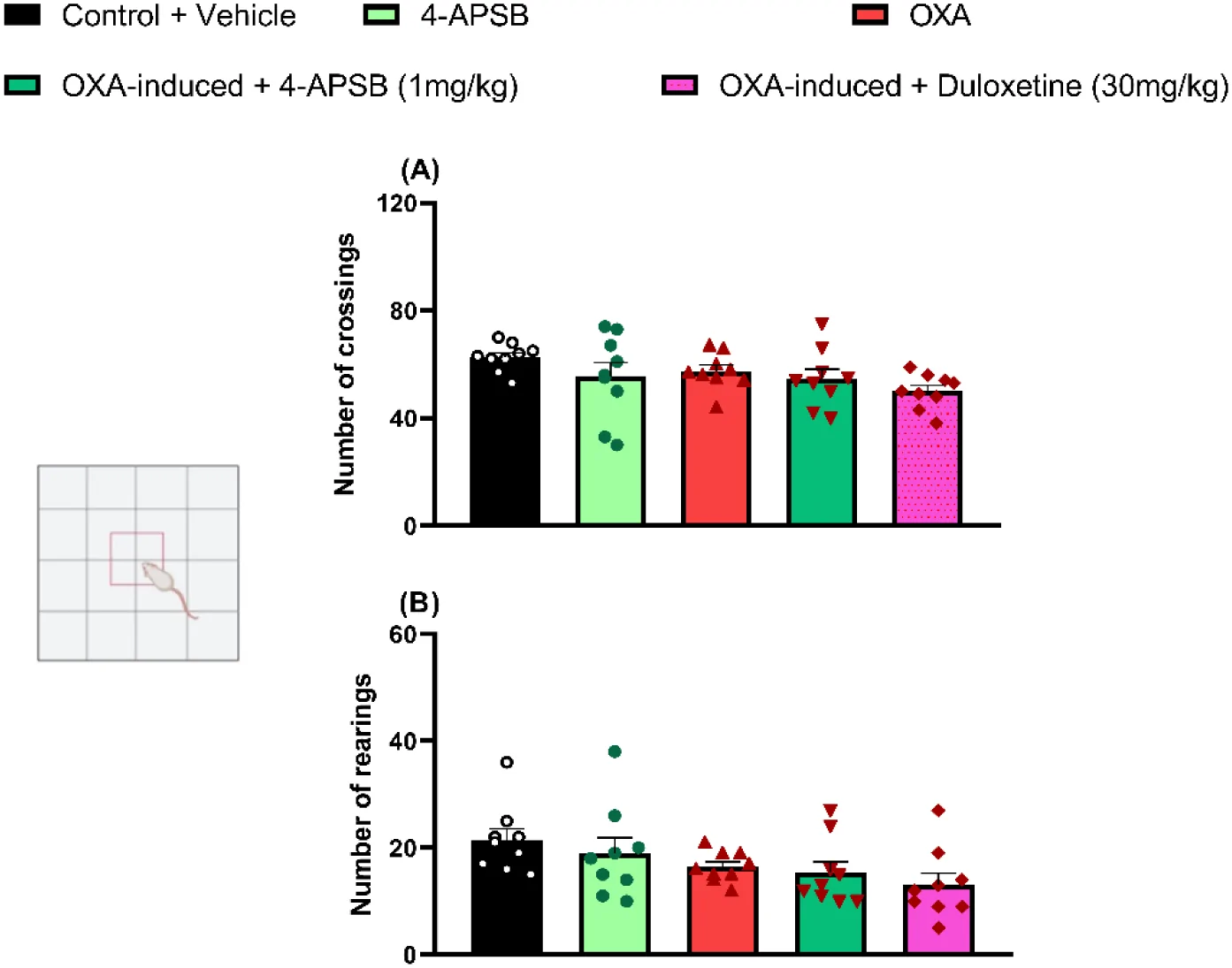
Effect of 4-amino-3-(phenylselanyl)­benzenesulfonamide (4-APSB) (1 mg/kg, i.g.) on the number of crossings (A) and the number of rearings (B) of female Wistar rats in the open field test. One-way ANOVA followed by Tukey’s test.

### 3.3 Na^+^/K^+^-ATPase, Mg^2+^-ATPase, and AChE Activities in OXA-Induced Acute Peripheral Neuropathy

The results presented in Figure 5 show that OXA administration inhibited Na^+^/K^+^-ATPase activity in both the cerebral cortex (Figure 5A) (ANOVA: F_(4, 40)_ = 41.42, *p* < 0.0001) and the spinal cord (Figure 5C) (ANOVA: F_(4, 39)_ = 8.969, *p* < 0.0001) of rats, compared with the control group. In addition, OXA-treated animals showed a significant increase in AChE activity in the cerebral cortex (Figure 5G) (ANOVA: F_(4, 40)_ = 10.22, *p* < 0.0001). Further, OXA did not cause significant changes in Mg^2+^-ATPase activity in any of the evaluated tissues (Figure 5D, 5E, and 5F).

**Figure 5:**
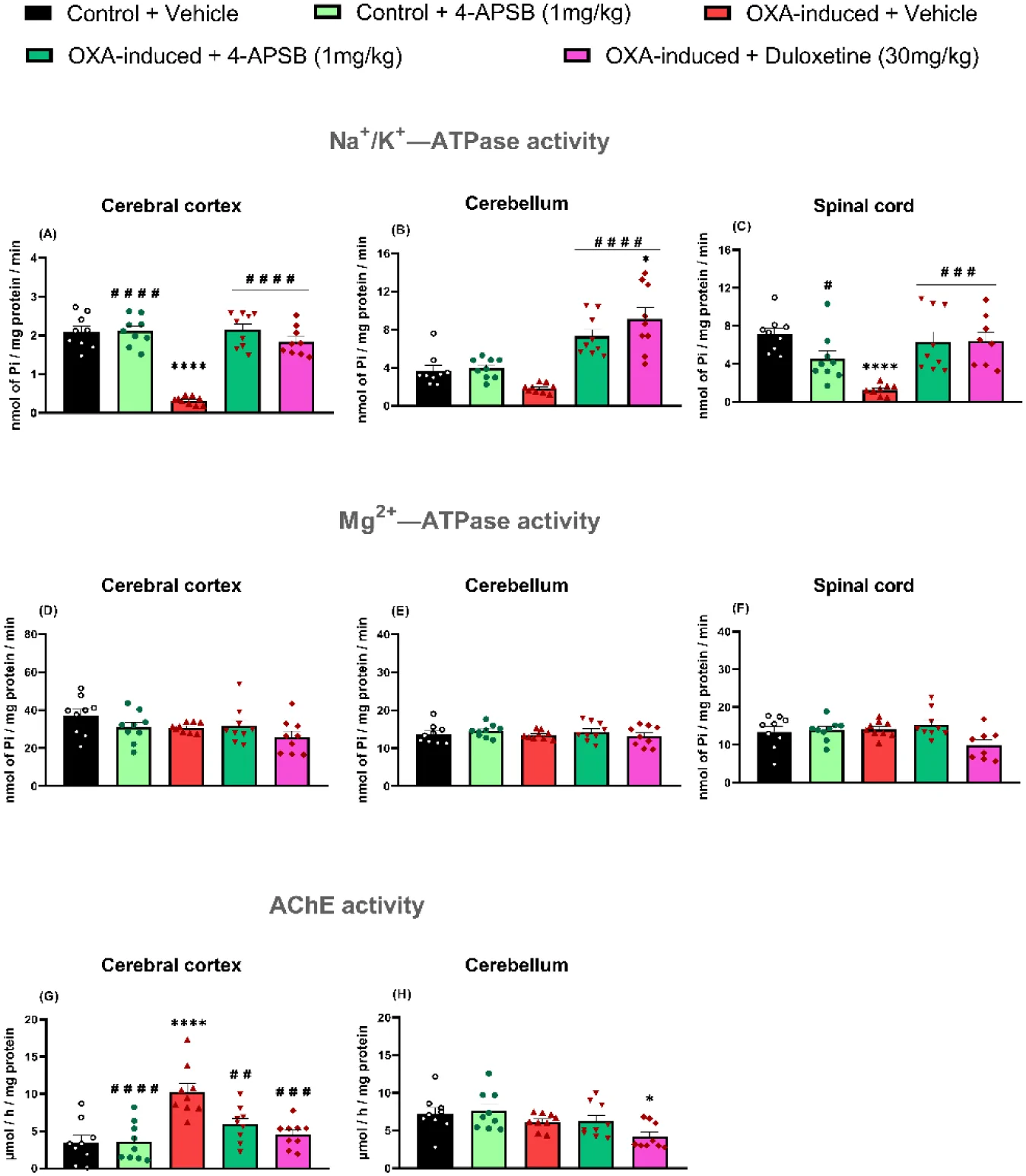
Effect of 4-amino-3-(phenylselanyl)­benzenesulfonamide (4-APSB) (1 mg/kg, i.g.) on Na^+^/K^+^-ATPase activity in the cerebral cortex (A), cerebellum (B), and spinal cord (C); on Mg^2+^-ATPase activity in the cerebral cortex (D), cerebellum (E), and spinal cord (F); and on AChE activity in the cerebral cortex (G) and cerebellum (H) in a model of oxaliplatin (OXA)-induced acute peripheral neuropathy in female Wistar rats. Asterisks indicate significance levels when compared with the control: (*) *p* < 0.05 and (****) *p* < 0.0001. (^#^) *p* < 0.05, (^##^) *p* < 0.01, (^###^) *p* < 0.001, and (^####^) *p* < 0.0001 indicate significance levels when compared with OXA. One-way ANOVA followed by Tukey’s test.

Duloxetine activated Na^+^/K^+^-ATPase in the cerebral cortex (Figure 5A), cerebellum (Figure 5B), and spinal cord (Figure 5C) samples compared with tissues from OXA-induced animals. Moreover, treatment with duloxetine restores AChE activity in the cerebral cortex (Figure 5G) relative to that of OXA-induced samples. Interestingly, OXA-induced rats treated with duloxetine showed activation of Na^+^/K^+^-ATPase (Figure 5B) and inhibition of AChE (Figure 5H) in cerebellar samples compared with the control group.

Treatment with 4-APSB (1 mg/kg), however, reversed the alterations caused by OXA. Specifically, it restored Na^+^/K^+^-ATPase activity (Figure 5A–C) in the cerebral cortex, cerebellum, and spinal cord, and AChE activity in the cerebral cortex (Figure 5G).

### 3.4 Oxidative Stress in OXA-Induced Acute Peripheral Neuropathy

#### 3.4.1 TBARS Levels

Figure 6 shows that OXA administration increased TBARS levels exclusively in the spinal cord compared with the control group (Figure 6C). Duloxetine induced lipid peroxidation in the cerebellum (Figure 6B) (ANOVA: F_(4, 40)_ = 2.860, *p* = 0.0356), a change not observed in OXA-induced rats, and failed to reduce the elevated TBARS levels in the spinal cord (Figure 6C). Similarly, 4-APSB (1 mg/kg) did not prevent the OXA-induced increase in spinal cord TBARS (Figure 6C) (ANOVA: F_(4,40)_ = 6.059, p = 0.0007).

**Figure 6:**
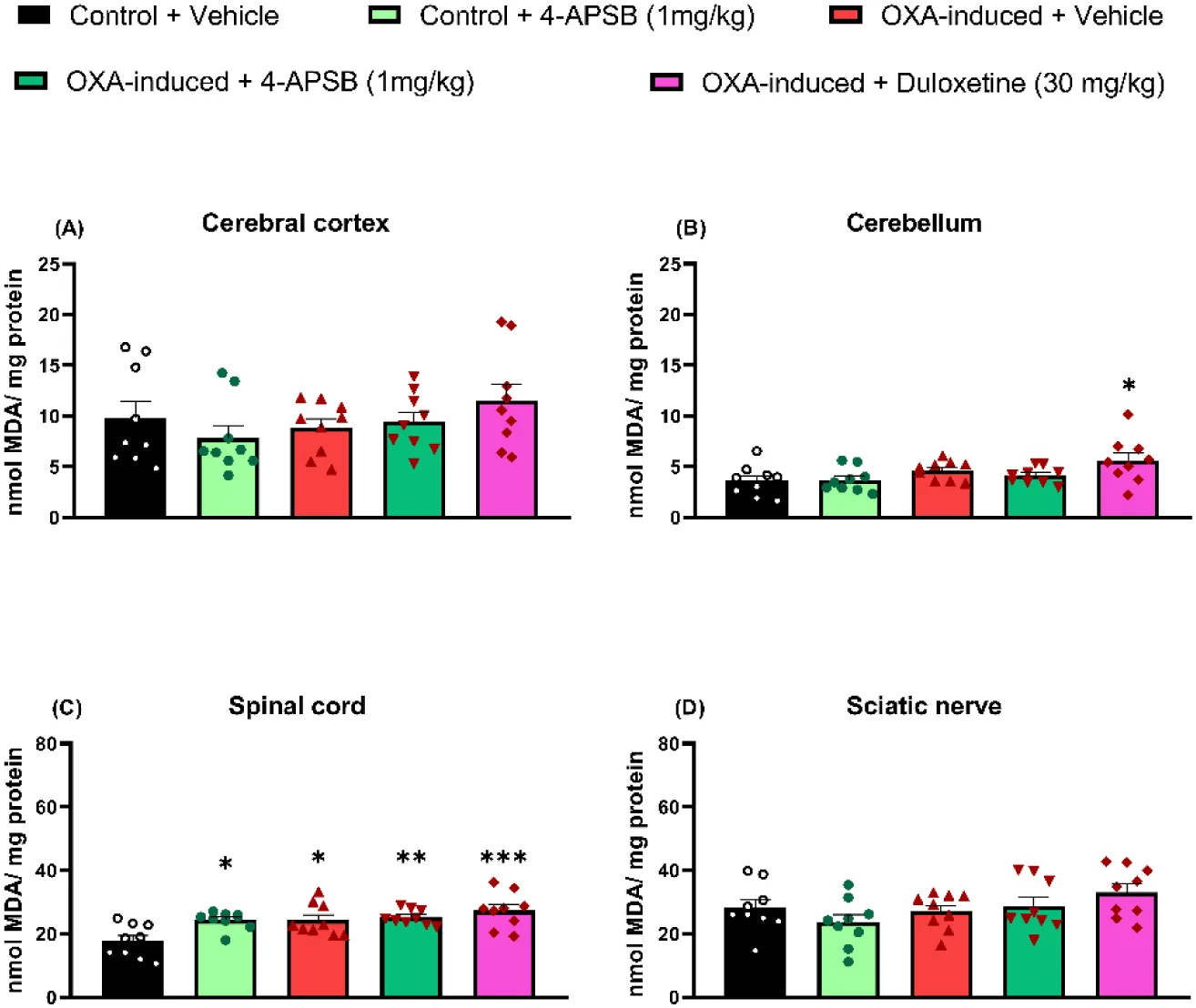
Effect of 4-amino-3-(phenylselanyl)­benzenesulfonamide (4-APSB) (1 mg/kg, i.g.) on levels of thiobarbituric acid reactive species (TBARS) in the cerebral cortex (A), cerebellum (B), spinal cord (C), and sciatic nerve (D) in a model of oxaliplatin (OXA)-induced acute peripheral neuropathy in female Wistar rats. Asterisks indicate significance levels when compared with the control: (*) *p* < 0.05, (**) *p* < 0.01, (***) *p* < 0.001, and (****) *p* < 0.0001. One-way ANOVA followed by Tukey’s test.

#### 3.4.2 RS Levels

The results presented in Figure 7 show that OXA administration caused an increase in RS levels in all tissues evaluated (cerebral cortex, cerebellum, spinal cord, and sciatic nerve) when compared with the control group (Figure 7A).

**Figure 7:**
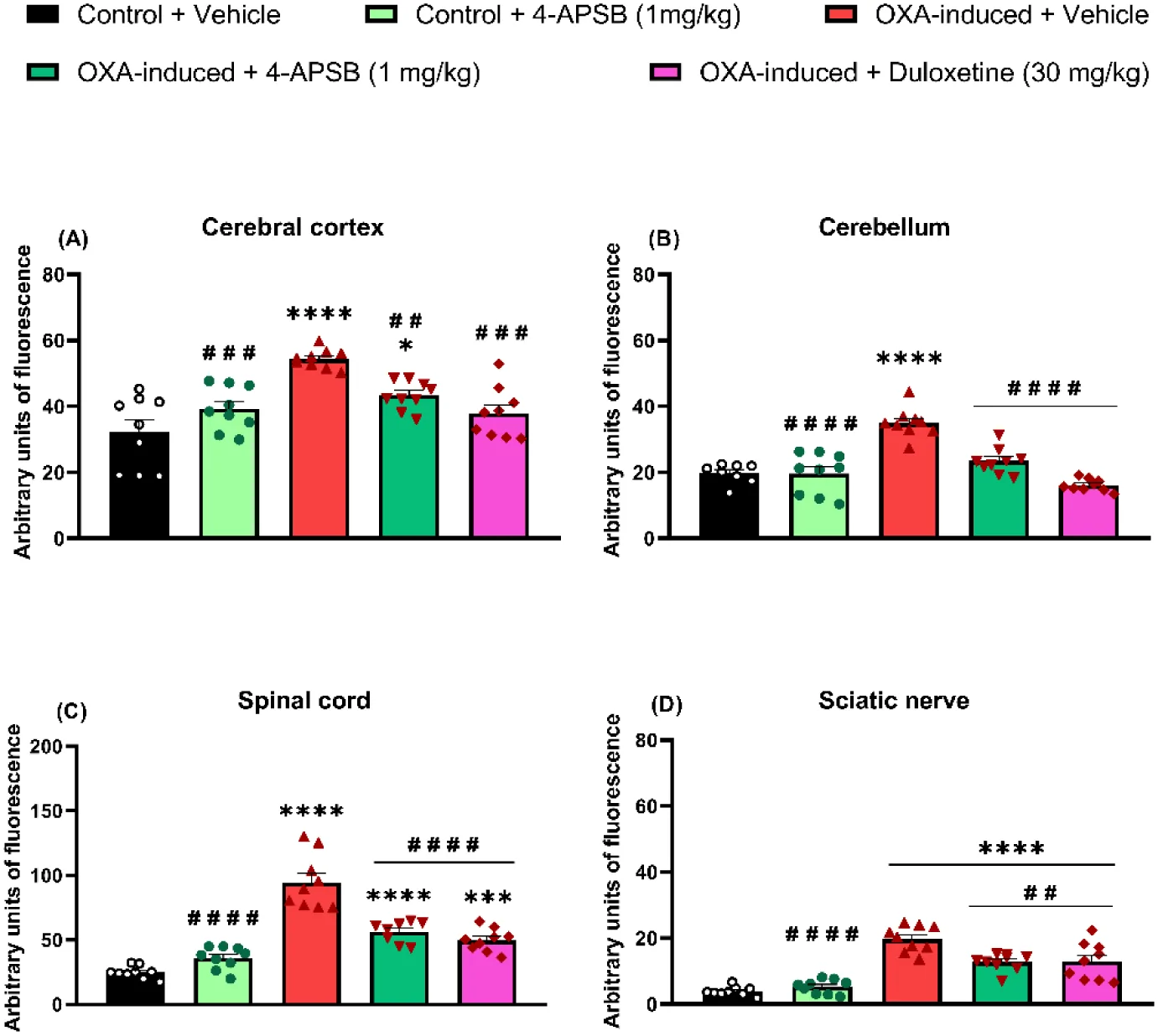
Effect of 4-amino-3-(phenylselanyl)­benzenesulfonamide (4-APSB) (1 mg/kg, i.g.) on levels of reactive species (RS) in the cerebral cortex (A), cerebellum (B), spinal cord (C), and sciatic nerve (D) in a model of oxaliplatin (OXA)-induced acute peripheral neuropathy in female Wistar rats. Asterisks indicate significance levels when compared with the control: (*) *p* < 0.05, (***) *p* < 0.001, and (****) *p* < 0.0001. (^##^) *p* < 0.01, (^###^) *p* < 0.001, and (^####^) *p* < 0.0001 indicate significance levels when compared with OXA. One-way ANOVA followed by Tukey’s test.

In contrast, duloxetine reduced RS levels in the cerebral cortex, cerebellum, spinal cord, and sciatic nerve (Figure 7A–D) following OXA induction. Similarly, 4-APSB (1 mg/kg) significantly decreased RS levels across all evaluated tissues (Figure 7A–D). (ANOVA: F_(4, 40)_ = 12.20, *p* < 0.0001) (Figure 7A); (ANOVA: F_(4, 40)_ = 27.62, *p* < 0.0001) (Figure 7B); (ANOVA: F_(4, 39)_ = 45.02, *p* < 0.0001) (Figure 7C); (ANOVA: F_(4, 40)_ = 30.59, *p* < 0.0001) (Figure 7D). Notably, duloxetine showed a superior effect in reducing RS levels in the cerebral cortex (Figure 7A) compared with 4-APSB.

#### 3.4.3 Nitrite and Nitrate (NOx) Levels

The results presented in Figure 8 show that OXA administration led to a significant increase in nitrite and nitrate levels in the cerebral cortex, spinal cord, and sciatic nerve (Figure 8A–D, respectively) compared with the control group, while no significant changes were observed in the cerebellum (ANOVA: F_(4, 40)_ = 4.963, *p* = 0.0024) (Figure 8A); (ANOVA: F_(4, 40)_ = 2.878, *p* = 0.0348) (Figure 8B); (ANOVA: F_(4, 40)_ = 10.94, *p* < 0.0001) (Figure 8C); (ANOVA: F_(4, 40)_ = 13.29, *p* < 0.0001) (Figure 8D).

**Figure 8:**
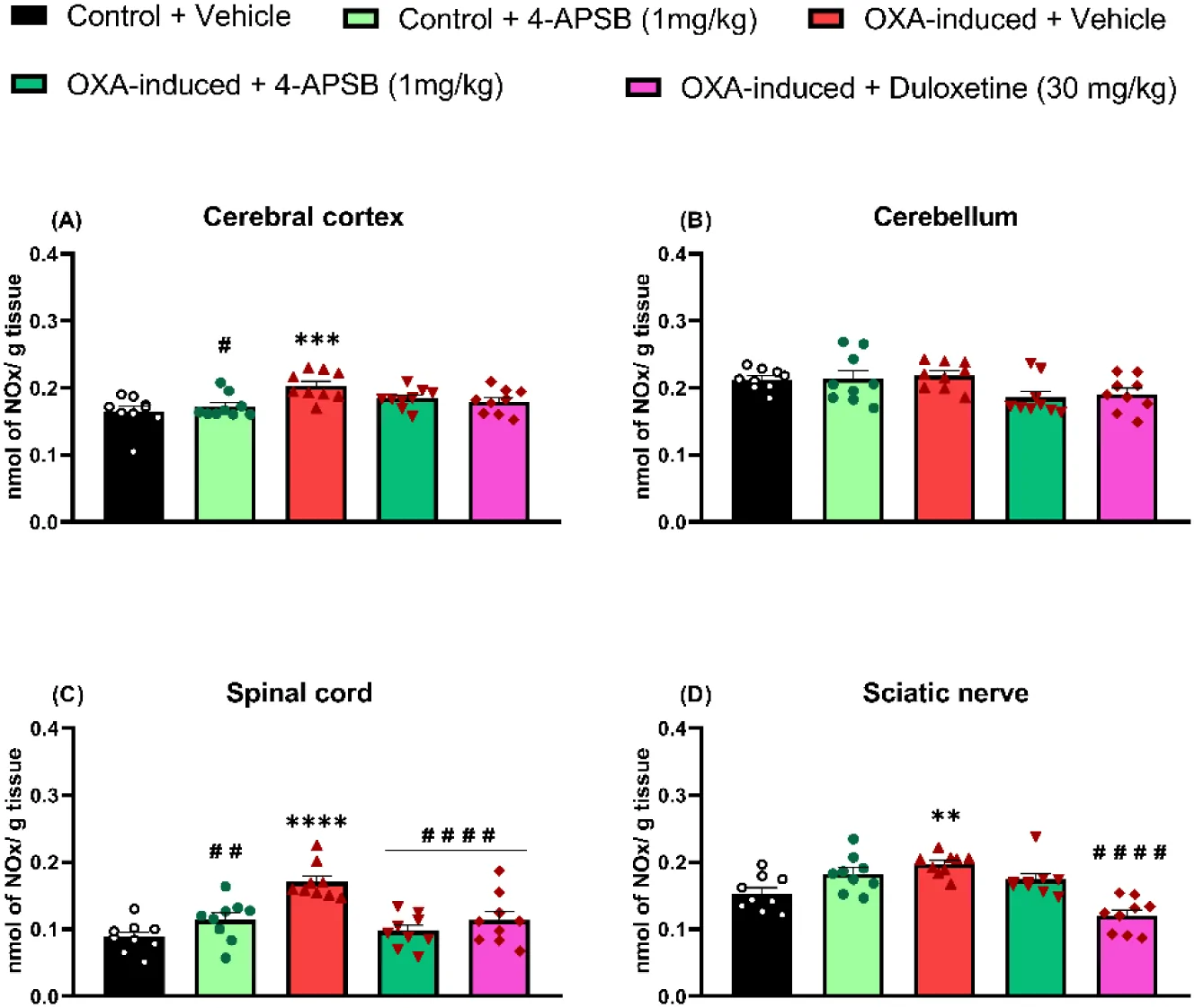
Effect of 4-amino-3-(phenylselanyl)­benzenesulfonamide (4-APSB) (1 mg/kg, i.g.) on nitrate and nitrite levels (NOx) in the cerebral cortex (A), cerebellum (B), spinal cord (C), and sciatic nerve (D) in a model of oxaliplatin (OXA)-induced acute peripheral neuropathy in female Wistar rats. Asterisks indicate significance levels when compared with the control: (**) *p* < 0.01, (***) *p* < 0.001, and (****) *p* < 0.0001. (^####^) *p* < 0.0001 indicate significance levels when compared with OXA. One-way ANOVA followed by Tukey’s test.

The increased levels of nitrite and nitrate were reversed with duloxetine treatment in the spinal cord (Figure 8C) and the sciatic nerve (Figure 8D). However, the treatment with 4-APSB (1 mg/kg) reversed the OXA-induced elevation in nitrite-nitrate levels exclusively in the spinal cord (Figure 8C) (ANOVA: F_(4, 40)_ = 10.94, *p* < 0.0001).

#### 3.4.4 CAT Activity

The results presented in Figure 9 show that OXA administration inhibited CAT activity in all evaluated tissues (cerebral cortex, cerebellum, spinal cord, and sciatic nerve) when compared with the control group (Figure 9A) (ANOVA: F_(4, 40)_ = 38.58, *p* < 0.0001); (Figure 9B) (ANOVA: F_(4, 40)_ = 24.78, *p* < 0.0001); (Figure 9C) (ANOVA: F_(4, 39)_ = 33.89, *p* < 0.0001); (Figure 9D) (F_(4, 40)_ = 16.87, *p* < 0.0001).

**Figure 9:**
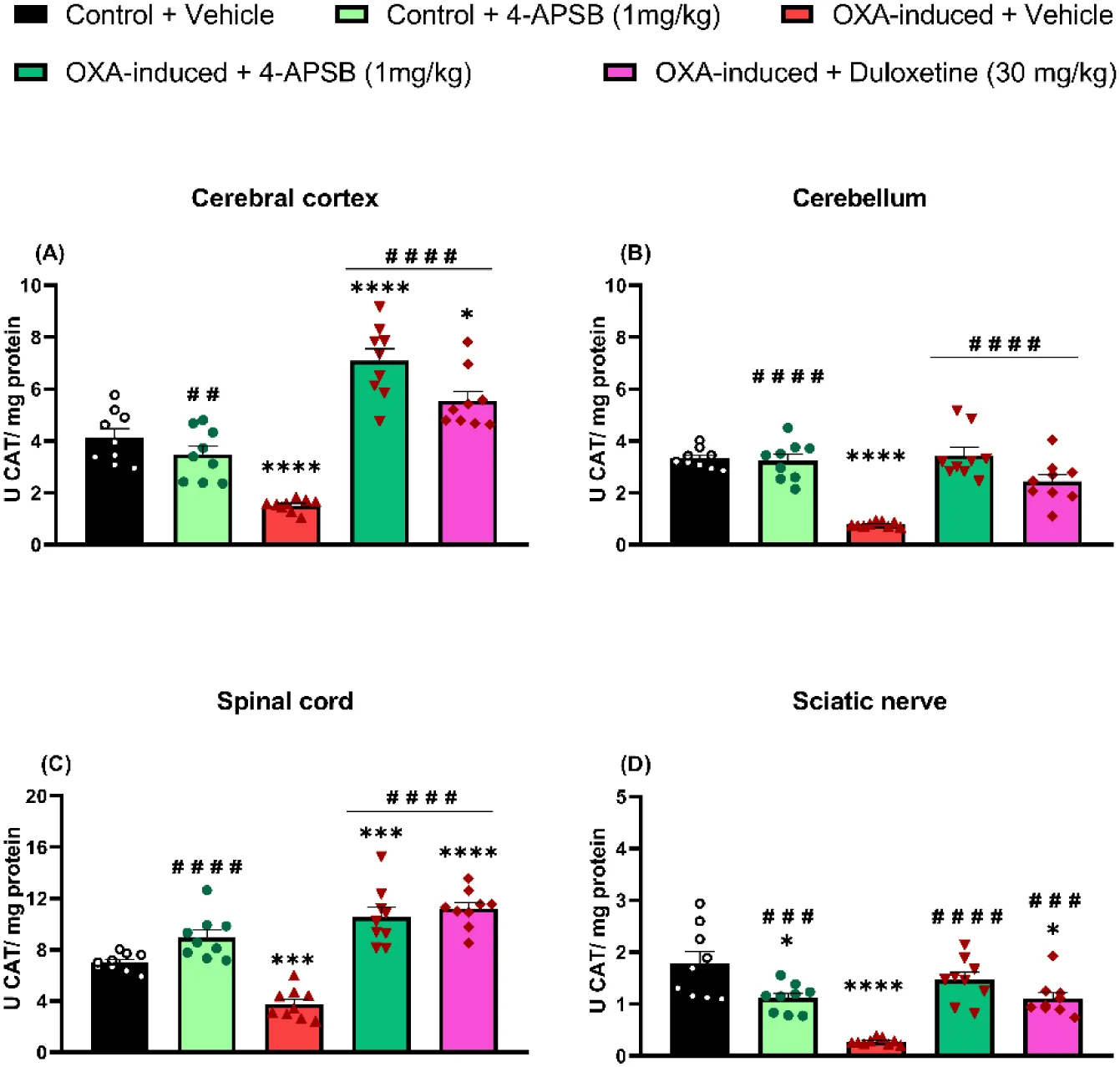
Effect of 4-amino-3-(phenylselanyl)­benzenesulfonamide (4-APSB) (1 mg/kg, i.g.) on catalase activity (CAT) in the cerebral cortex (A), cerebellum (B), spinal cord (C), and sciatic nerve (D) in a model of oxaliplatin (OXA)-induced acute peripheral neuropathy in female Wistar rats. Asterisks indicate significance levels when compared with the control: (*) *p* < 0.05, (***) *p* < 0.001, and (****) *p* < 0.0001. (^###^) *p* < 0.001, and (^####^) *p* < 0.0001 indicate significance levels when compared with OXA. One-way ANOVA followed by Tukey’s test.

Treatment with duloxetine restored CAT activity in all tissues evaluated (Figure 9A–D) compared with the OXA-induced group. Moreover, duloxetine increased CAT activity beyond control levels in the cerebral cortex (Figure 9A), spinal cord (Figure 9C), and sciatic nerve (Figure 9D) of animals exposed to OXA.

Treatment with 4-APSB (1 mg/kg) restored CAT activity in all tissues (Figure 9A–D) compared with that of OXA-induced samples. Interestingly, CAT activity in the cerebral cortex (Figure 9A) and spinal cord (Figure 9C) was higher than that in the control group. 4-APSB, *per se*, inhibited sciatic nerve CAT activity compared with the control group.

#### 3.4.5 NPSH Levels

The results presented in Figure 10 show that OXA administration did not cause significant changes in NPSH levels in the cerebral cortex, spinal cord, and sciatic nerve when compared to the control group (Figure 10A) (ANOVA: F_(4, 40)_ = 0.7631, *p* = 0.5556); (Figure 10B) (ANOVA: F_(4, 40)_ = 8.859, *p* < 0.0001); (Figure 10C) (ANOVA: F_(4, 39)_ = 3.999, *p* < 0.0080). Similarly, treatment with 4-APSB also did not significantly alter NPSH levels in the tissues evaluated (Figure 10A–C).

**Figure 10:**
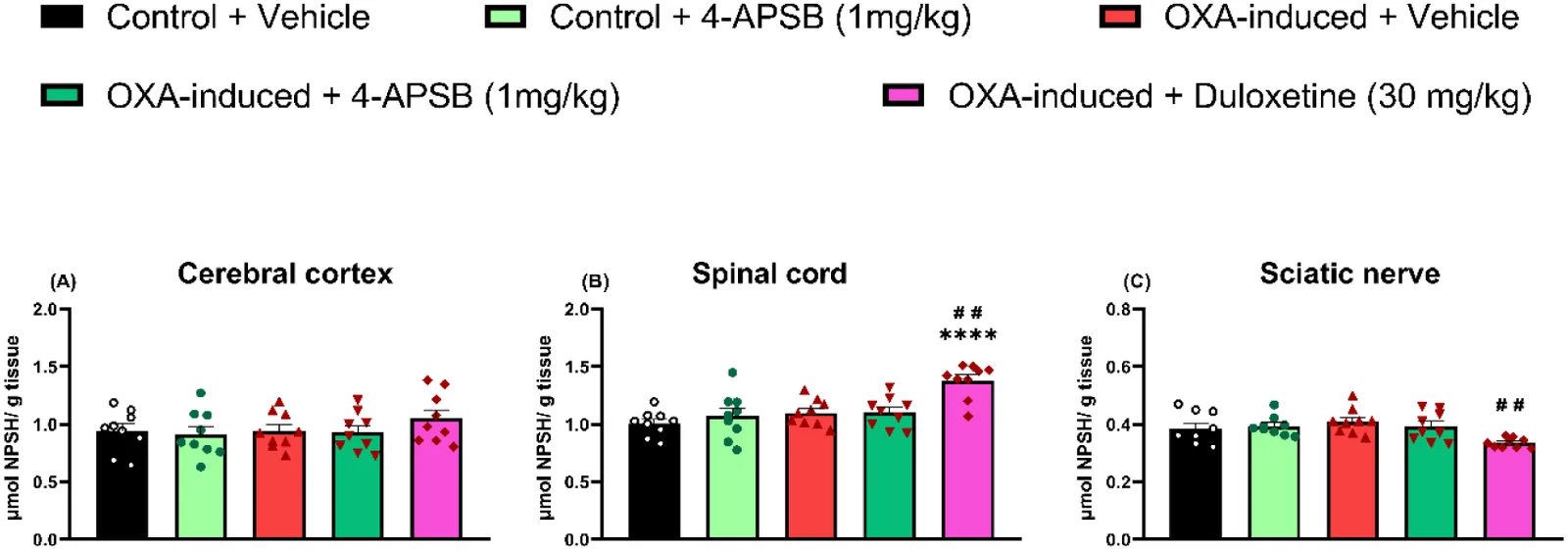
Effect of 4-amino-3-(phenylselanyl)­benzenesulfonamide (4-APSB) (1 mg/kg, i.g.) on levels of nonprotein thiols (NPSH) in the cerebral cortex (A), spinal cord (B), and sciatic nerve (C) in a model of oxaliplatin (OXA)-induced acute peripheral neuropathy in female Wistar rats. Asterisks indicate significance levels when compared with the control: (****) *p* < 0.0001 and (^####^) *p* < 0.0001 indicate significance levels when compared with OXA. One-way ANOVA followed by Tukey’s test.

Despite this, duloxetine treatment increased NPSH levels in spinal cord tissue compared to both the control and OXA-induced groups (Figure 10B). In contrast, in the sciatic nerve (Figure 10C), duloxetine reduced NPSH levels relative to those of the OXA-induced group.

## 4 Discussion

This study expands the understanding of the pharmacological effects of organoselenium compounds, showing that 4-APSB interferes with multiple critical pathways involved in the pathophysiology of OXA-induced peripheral neuropathy in rats. We investigated the potential of 4-APSB in an experimental model of OXA-induced acute peripheral neuropathy, evaluating its ability to modulate nociceptive behavior and reverse biochemical alterations associated with oxidative stress and enzymatic dysfunction. The results demonstrated that 4-APSB reduced OXA-induced pain hypersensitivity without affecting spontaneous locomotion. Moreover, the compound attenuated oxidative alterations, reduced protein damage, and normalized antioxidant enzyme activity. Restoration of Na^+^/K^+^-ATPase and AChE activities in the cerebral cortex was also observed. Taken together, these findings indicate that 4-APSB combines antinociceptive and antioxidant properties, providing both functional and biochemical protection against OXA-induced neurotoxicity.

Furthermore, this study contributes to the broader field of toxicological sciences by characterizing the specific neuroenzymatic and redox signatures of OXA-induced acute injury. While the focus remains on the protective potential of 4-APSB, our findings emphasize that the disruption of Na^+^/K^+^-ATPase activity and the induction of systemic oxidative stress are fundamental markers of OXA’s toxicological profile across the peripheral and central nervous systems. Identifying these emerging toxicological targets, specifically the Na^+^/K^+^ pump and AChE activity, provides a clearer understanding of the biochemical triggers for neuronal hyperexcitability and sensory dysfunction. Therefore, the ability of 4-APSB to normalize these parameters not only demonstrates its therapeutic promise but also validates these specific pathways as critical intervention points for mitigating the neurotoxicological burden and dose-limiting adverse effects of OXA.

Peripheral neuropathy is a clinically relevant and dose-limiting complication that often compromises patient adherence to oncological treatment, impairing the quality of life.^1,23^ It presents two distinct components: the acute form, which manifests within hours or a few days after infusion and is characterized by cold allodynia, hyperalgesia, and transient paresthesia; and the chronic form, which arises from cumulative exposures and is marked by distal axonal degeneration and persistent symptoms.^1,24^ The present preclinical study in an animal model has deepened our understanding of the pathophysiological mechanisms underlying OXA-induced acute peripheral neuropathy and provided relevant advances in its potential therapeutic management.

Our research group has been investigating preclinical adverse effects of OXA across different contexts, aiming to deepen understanding of its pathophysiological mechanisms and identify therapeutic strategies. In a recent study, we demonstrated that aging increases pain sensitivity in mice exposed to OXA, which was associated with enhanced oxidative damage and inhibition of Na^+^/K^+^-ATPase, mechanisms directly related to the greater vulnerability of aged animals to neuropathy development.^6^ In other investigations, we explored the involvement of the cholinergic pathway and showed that OXA not only induces peripheral oxidative stress but also crosses the blood–brain barrier, accumulates in the central nervous system, and triggers neurotoxicity.^5,25,26^ We have also examined OXA-induced hepatotoxicity and renal injury.^7,27^

In this context, we further demonstrated that organoselenium compounds exert antioxidant effects that restore OXA-induced redox imbalance in both the peripheral and central nervous systems, thereby reducing pain sensitivity.^5,6,26^ Collectively, these findings highlight the importance of investigating the mechanisms underlying OXA-induced neurotoxicity and support the pharmacological potential of organoselenium compounds as therapeutic alternatives. In the present study, we sought to address a limitation of previous investigations by evaluating pathophysiological mechanisms in a female animal model, thereby broadening the analysis to include biological sexes—an essential aspect for translational relevance and comprehensiveness of the results.

Although considerable research efforts have been made, no widely accepted safe and effective therapies are currently available for CIPN.^8^ Therefore, the search for agents with protection against acute neurotoxicity or disease-modifying properties remains a scientific and clinical priority. Despite decades of use of OXA in oncology, prophylactic and therapeutic strategies are still lacking. Duloxetine is the intervention with the most consistent benefit; however, its efficacy is only moderate and subject to substantial interindividual variability.^8^ In this context, compounds with redox-active properties and the ability to modulate neuronal excitability, such as organoselenium derivatives, emerge as promising candidates to attenuate not only the acute phase but also the mechanisms that predispose to the chronicity of neuropathy.^5,6,26^

The alleviation of OXA-induced hypersensitivity by 4-APSB is consistent with evidence identifying oxidative stress and mitochondrial dysfunction as central drivers of OXA-induced neuropathic pain,^24^ particularly in cold- and mechanical-Aδ and C sensory fibers.^28^ Preclinical studies with organoselenium compounds, including 4-APSB, have shown GPx-mimetic activity, reduction of lipid peroxidation, and preservation of membrane proteins—effects aligned with the damage-limiting profile observed in the present study.^29,30^ Moreover, in a study conducted by our group, 4-APSB exhibited antinociceptive effects in acute nociception models through its antioxidant action,^9^ reinforcing the mechanisms proposed herein. The behavioral improvement observed with 4-APSB, without impairing locomotor activity, supports a specific antinociceptive effect rather than a sedative or motor effect, a key feature for the translational potential of any candidate analgesic or antineuropathic drug.

On the biochemical axis, OXA treatment increased oxidative damage, a pattern widely described in both acute and chronic models of OXA-induced neuropathy.^24,31^ Treatment with 4-APSB reversed these alterations by reducing the levels of markers of oxidative damage and restoring antioxidant defenses. The literature recognizes that the vulnerability of sensory neurons to OXA is partly attributable to their high bioenergetic demand and to the susceptibility of the axonal sheath and ion channels to oxidative stress.^24,31^ Thus, mitigating redox damage appears to be a prerequisite for behavioral benefits to emerge, precisely as observed in the present study.

Regarding the neuroenzymatic axis, treatment with OXA impaired Na^+^/K^+^-ATPase activity, which is essential for maintaining the ionic gradient and membrane potential, thereby directly determining excitability thresholds and nerve conduction.^32,33^ Restoration of this activity by 4-APSB provides a plausible explanation for the observed reduction in hypersensitivity, as the preservation of ATPase function helps stabilize neuronal excitability and prevent hyperexcitability in peripheral and central nociceptive circuits. The concomitant modulation of AChE in brain tissue further suggests that 4-APSB influences cholinergic homeostasis. Although muscarinic or nicotinic receptors were not directly assessed, it is reasonable to assume that restoring AChE activity to physiological levels promotes fine-tuning of cholinergic tone, an axis known to participate in spinal and supraspinal antinociceptive mechanisms.^34^ Unlike drugs that act as downstream neurotransmitter modulators, correction of proximal determinants such as redox balance, bioenergetics, and membrane integrity may reduce the likelihood that acute sensitization progresses to chronicity. While this remains hypothetical until validated in cumulative models, it represents a promising direction.

The integration of these axes suggests a composite mechanism comprising three layers. The first is antioxidant, consistent with the chemistry of organoselenium compounds.^30^ By acting on peroxides and preserving GSH, 4-APSB reduces the propagation of lipid and protein damage in membranes, including microdomains that house pumps and ion channels. The second is bioenergetic, through the functional protection of ATPases. In sensory neurons and myelinated fibers, Na^+^/K^+^-ATPase is a major consumer of ATP. Thus, its dysfunction not only increases excitability but also promotes cycles of calcium overload and additional production of reactive species.^32,33^ The restoration of this pump suggests that 4-APSB helps reduce membrane hyperexcitability. The third layer is cholinergic, via modulation of AChE. Alterations in acetylcholine catabolism can affect inhibitory interneurons and descending pain modulation circuits.^34^ The normalization of AChE activity by 4-APSB, as observed in the present study, is consistent with the reduction of the central nociceptive gain. However, it must be acknowledged that while the restoration of redox, ionic, and cholinergic homeostasis correlates with the observed behavioral improvements, the present experimental design does not definitively establish a causal link. Therefore, these pathways should be regarded as potential mechanisms that may also occur secondary to an overarching reduction in oxidative stress.

It is noteworthy that duloxetine also attenuated hypersensitivity, as expected from its dual mechanism of serotonin and norepinephrine reuptake inhibition and consequent facilitation of descending inhibitory pathways.^35^ However, a more detailed analysis reveals effects that raise concerns about its pharmacological profile. Duloxetine increased the latency for thermal sensitivity perception beyond control values, indicating an alteration of the basal pain perception. Since nociception plays a fundamental role in physiological defense, this finding suggests a potentially deleterious effect by impairing the detection of noxious stimuli.

In the cerebellum, the biochemical response to duloxetine also differed from that of the control animals. Na^+^/K^+^-ATPase activity was elevated above basal levels, while AChE activity was significantly reduced. These changes indicate an atypical modulation of cholinergic neurotransmission and ionic homeostasis, suggesting that although duloxetine is effective in relieving hypersensitivity, it may also induce functional imbalances in the cerebellar circuits. Furthermore, the combination of duloxetine with OXA increased lipid peroxidation levels in the cerebellum, whereas OXA alone or coadministration with 4-APSB did not change lipid peroxidation levels. This finding suggests that the pharmacological combination may potentiate oxidative damage, adding a dimension of risk to its use under the conditions of OXA-induced neuropathy. Collectively, these results underscore the need for a critical evaluation of duloxetine use in the management of OXA-induced peripheral neuropathy, as its adverse effects on basal nociception, neurotransmission, and redox balance may limit its clinical applicability and compromise long-term safety.

Conversely, the biochemical normalization observed with 4-APSB, particularly regarding oxidative stress and ATPases, suggests a potential disease-modifying effect that goes beyond simple symptomatic analgesia. Although the present study does not address the cumulative, chronic component of peripheral neuropathy, the restoration of the redox environment and membrane function could, in theory, reduce the transition from the acute to the chronic phase—a hypothesis to be tested in repeated OXA exposure protocols. Administration of 4-APSB *per se* did not alter nociceptive behavior or most of the biochemical markers analyzed, supporting a short-term functional safety profile and minimizing the probability of pronociceptive or pro-oxidative effects. Furthermore, the absence of locomotor interference indicates a favorable therapeutic window, an important consideration for adjuvant agents in oncology, where sedation and motor impairment must be avoided to preserve patient autonomy. Although the present study demonstrates promising antinociceptive and biochemical effects of 4-APSB in OXA-induced acute peripheral neuropathy, its potential influence on the antitumor efficacy of OXA remains to be fully elucidated. Because oxidative stress contributes, at least in part, to the cytotoxic activity of platinum-based chemotherapy, antioxidant therapies may theoretically interfere with the anticancer efficacy. To address this important issue, studies in our laboratory using tumor-bearing experimental models are currently underway to investigate whether 4-APSB affects tumor progression, tumor cell viability, or OXA-mediated cytotoxicity. These ongoing investigations will provide essential information regarding the safety and translational potential of 4-APSB as an adjuvant therapy for cancer treatment.

In summary, the data from the present study indicate that 4-APSB has a beneficial multitarget profile for treating peripheral neuropathy. It reduces hypersensitivity without motor impairment, corrects oxidative stress, restores ATPases critical for neuronal electrical stability, and modulates AChE in a manner that is compatible with analgesia. These findings demonstrate that 4-APSB exerts antinociceptive and antioxidant effects in OXA-induced acute peripheral neuropathy, reversing biochemical and functional alterations associated with redox imbalance and enzymatic dysfunction. Notably, this investigation was conducted in a female animal model, overcoming a limitation of previous studies, which were predominantly performed in males, thereby broadening the applicability of the findings across biological sexes.

It should be emphasized that the focus of this work was to elucidate pathophysiological mechanisms and explore therapeutic alternatives for acute OXA-induced neuropathy. Extrapolations to chronic and cumulative neuropathy will require new experimental models and additional studies. Nevertheless, the results presented here reinforce the potential of organoselenium compounds as promising pharmacological alternatives, capable of addressing an unmet therapeutic need in the management of acute OXA-induced peripheral neuropathy. Although the present findings provide consistent evidence for the acute antinociceptive effects and associated biochemical modulation produced by 4-APSB in an experimental model of acute OXA-induced peripheral neuropathy, several limitations should be acknowledged. First, the acute OIPN model does not reproduce the cumulative and persistent manifestations of chronic chemotherapy-induced peripheral neuropathy, thereby limiting the extrapolation of our findings to chronic OIPN and long-term treatment conditions. Pharmacokinetic and tissue-distribution studies of 4-APSB were not performed. In addition, histological and electrophysiological analyses were not included in the present study, thereby precluding the assessment of structural alterations, axonal integrity, and nerve conduction, as well as their relationship with the observed behavioral and biochemical changes. Furthermore, only the 1 mg/kg dose of 4-APSB was evaluated. This dose was selected based on the promising antioxidant and antinociceptive effects previously reported for other organoselenium compounds. Given the substantial number of animals required for the behavioral and biochemical analyses, the present proof-of-concept study prioritized the evaluation of a single evidence-informed dose in accordance with the principles of the 3Rs, particularly Reduction. Nevertheless, this design did not allow the establishment of a dose–response relationship or the identification of the minimum effective and optimal doses. Finally, because tumor-bearing animals and tumor-related outcomes were not included, the present study cannot determine whether 4-APSB affects tumor progression or interferes with the antitumor activity of OXA.

The present study represents an initial step in the preclinical investigation of 4-APSB for the management of OXA-induced acute peripheral neuropathy. The observed attenuation of behavioral hypersensitivity and associated biochemical alterations warrants further investigation of this compound in more translational experimental settings. In this regard, our research group is currently conducting long-term studies using chronic neuropathy and tumor-bearing experimental models to evaluate the effects of repeated 4-APSB administration under conditions that more closely resemble the clinical scenario. These ongoing studies also include histological assessments and evaluations of whether 4-APSB influences tumor progression or the antitumor activity of OXA. Additional dose–response, pharmacokinetic, tissue distribution, and long-term safety studies will also be required. Collectively, these investigations are expected to provide a more comprehensive characterization of the pharmacological effects and potential mechanisms associated with 4-APSB and to help define its translational feasibility as a potential adjuvant for the management of OXA-induced peripheral neuropathy.

## Data Availability

All data sets generated and analyzed during this study are included in this article. Materials are available upon request.

## Notes

**Ethics Approval**: Animal care and all experimental procedures were conducted in compliance with the National Institute of Health Guide for the Care and Use of Laboratory Animals (NIH publication no. 80-23, revised in 1996) and in accordance with the Committee on Care and Use of Experimental Animal Resources, Federal University of Pelotas, Brazil (CEUA-UFPel) (23110.050294/2018-14). All efforts were made to minimize the number of animals used and their suffering. During the preparation of this work, the authors used GPTchat (OpenAI, 2025) to improve readability and language. After using this tool, the authors have took and edited the content as necessary and take full responsibility for the content of the publication.
